# Book Review

**Published:** 1902-09

**Authors:** 


**The Pocket Gray.** By the late Edward Cotterell. Fifth Edition. Edited by C. H. Fagge, M.B. Pp. 269. London: Baillière, Tindall & Cox. 1901.—As the title is intended to imply, the dimensions of this volume are such that it may be carried in the pocket. The pocket used must be a coat-pocket to avoid discomfort, and to enable the bearer to bring the book to the light of day easily and therefore frequently. That a fifth edition of this *vade mecum* has been required argues well for its popularity. It is certainly a *multum in parvo*. The condensation of the information given is so great that any prolonged attempt to read it may be expected to produce fatigue, and to lead to the reposition of the volume in the pocket from whence it came. We consider the book is well adapted to play the part of a refresher to the fading anatomical memory—and who is not aware of the ineradicable tendency of the anatomical memory to fade? There is no royal road to learning, but the way of repetition is a good one. Such a book as this facilitates repetition. It is also handy for rapid reference. If not used to the exclusion of larger works, we can emphatically recommend it to those who have to read anatomy while they run.

**The Medical Annual.** Bristol: John Wright & Co. 1902.—This is the twentieth year of issue of the *Medical Annual*, and the publishers state that the increased demands of their readers have led them to gradually increase the size of the volume, so that it is now three times as large as at first. It has been found that the general practitioner of to-day demands information on the very latest questions of medical science, that he is even interested, for example, in the details of operations which only specialists are likely to be called upon to perform. Part I. of the *Annual*—"The Dictionary of Materia Medica and Therapeutics"—is a review of therapeutic progress in the year 1901, and has been written by Dr. William Murrell. Prof. McFarland, of Philadelphia, assisted in writing the valuable article on toxins and antitoxins in this portion of the book. The major part of the volume is the dictionary of medicine and surgery, and the various articles there are from the pens of well-known authors and give much valuable information. They also supply useful references. Part III.—"Miscellaneous"—is concerned with sanitary science, legal matters and cases, new inventions, and new pharmaceutical and dietetic preparations, and provides lists of the books of the year, of lunatic asylums, of homes for inebriates, of training and nursing institutions, &c., &c. We are of the opinion that the *Medical Annual* well deserves an easily accessible place on the desk or book-shelf of every practitioner.

**Burdett's Hospitals and Charities.** London: The Scientific Press, Limited. 1902.—The value of this indispensable guide to the hospitals and philanthropic institutions all over the world is further enhanced in this volume by the addition of a carefully-selected list of sanatoria for the open-air treatment of cases of tuberculosis. Everybody interested in hospital work should make a point of reading the opening chapters of the book, those on the King's Coronation gift, hospital finance, and the nursing department and its cost.

**Transactions of the Royal Academy of Medicine in Ireland.** Edited by Dr. JOHN B. STORY. Vol. XIX. Dublin: Fannin and Co., Limited. 1902.—Dr. Sinclair gives a summary of two years of open-air treatment of consumption in Ireland, and many other papers on the usual variety of topics follow.

**Medico-Chirurgical Transactions.** Vol. LXXXIV. London: Longmans, Green and Co. 1901.—The most noteworthy papers of this volume relate to Phthisis—the one on "Metabolism in Phthisis," by Drs. Goodbody, Bardswell and Chapman; the other on "Wind Exposure and Phthisis," by Dr. W. Gordon. The President's address contains obituary notices of many well-known men, including Dr. William Marcet, Sir William Overend Priestley, Professor George Viner Ellis, Dr. Julius Althaus, Dr. Daniel John Leech, Sir William Stokes, Sir Henry Wentworth Dyke Acland, Bart., K.C.B., and Arthur Symons Eccles. The recent advances in the knowledge of malaria are described by Dr. Patrick Manson, and experiments in the mosquito-malaria theory by Drs. Sambon and Low.

**Transactions of the College of Physicians of Philadelphia.** Third Series. Vol. XXIII. Philadelphia: Printed for the College. 1901.—This volume is worthy of especial attention for its two memoirs—the one of Sir James Paget, written by Dr. J. C. Da Costa; the other of Dr. Wm. Pepper, by Dr. James Tyson. From the former we quote the following:—"Paget was an Englishman, we are Americans; but we claim a place among the mourners at his tomb, for no fame can rise and burn in England but that it reddens these western skies. The lines which divide us are traced in water; the bonds which unite us are stronger than steel; and such men as Sir James Paget do more to bring nations into brotherhood than do sworn compacts and written treaties. . . . He was a great man, a good man, a gentleman. Peace to his soul."

**Annual Report of the Smithsonian Institution, 1900.** Washington: Government Printing Office. 1901.—As is only to be expected, a good many of the papers in this volume are retrospects of the progress in the various branches of science during the nineteenth century, all of them written by most distinguished men of science in both hemispheres. It is only when one is brought face to face with facts collected together, as in the present Report, that one realises the enormous strides that have been made in scientific knowledge, and the consequent benefit to mankind, in the space of a hundred years. Take electricity for instance. Prof. Elihu Thomson's paper reads like a romance. Amongst the papers of more interest to the general reader, we would draw attention to Ewart S. Grogan's account of his travels "Through Africa from the Cape to Cairo";

"Nature Pictures," by A. Radclyffe Dugmore; and, more especially, Count D'Hérisson's account of "The Loot of the Imperial Summer Palace at Pekin." This most graphic story is published on account of its "great ethnological value, and for its apparently fair account of the mental processes of the Chinese ruling classes and their attitude towards foreigners, and the frank statement of the uncontrollableness and barbarism into which trained European soldiers may relapse under temptation." The medical papers include Dr. Billing's address on the "Progress of Medicine in the Nineteenth Century"; a summary of our present knowledge of "Malaria," by Dr. Sternberg; and a paper on the "Transmission of Yellow Fever by Mosquitoes," by the same author. We cannot help thinking that a volume containing so much valuable material is worthy of a better and more substantial binding.

**Anæsthetics and their Administration.** By FREDERICK W. HEWITT. [Second Edition.] Pp. xxiv., 528. London: Macmillan and Co., Limited. 1901.—This book as now published is substantially a new one, and forms a most complete work on everything relating to anæsthetics. In addition to a chapter on the evolution of anæsthetics, we have other chapters treating of such subjects as the properties and impurities of the chief agents used, and the theoretical and experimental physiology of general surgical anæsthesia. Then we have the general principles to be observed in selecting anæsthetics and the various methods of administering them, while full details are added of the individual peculiarities of each separate anæsthetic. The author considers that the much disputed point whether chloroform has or has not a directly depressing action on the heart must now be regarded as settled in the affirmative. He advances so many good reasons for this belief, and so well sums up the Hyderabad controversy, that we have no doubt his decision will meet with the approval of almost all readers. The author considers that, next to nitrous oxide, ether is the safest anæsthetic; and here again he will be approved by most surgeons, although the Scotch school and the followers of Dr. Laurie will doubtless remain of their own opinion still. We are glad to notice that Dr. Hewitt recommends the administration of ether in prolonged dental operations when nitrous oxide is insufficient, but that he considers chloroform the best anæsthetic to use for parturition. He considers the clinical fact established beyond doubt that in surgical practice the best subjects for chloroform are those whose general vital functions are somewhat impaired by illness; while powerful, muscular, young men often take it badly. There has been much talk in some circles lately about septic pneumonia having been set up by the use of foul inhalers, and we notice that Dr. Hewitt is very particular about cleanliness. He says it would be impossible, even were it advisable, to sterilize, after each administration, the mouth-pieces, bags, &c., employed; but it should be a matter of routine to well wash in hot water everything that has been used. This book is the most complete on the subject of anæsthetics that we have seen. It is full of information and good sense, and we have pleasure in recommending it.

**Manipulation (or Massage).** By John Andrew Peters. Pp. xvi., 159. Newcastle-on-Tyne: Longhurst. 1901.—This thesis is the outcome of twenty years' practical work and observation of "manual treatment" by one who has not aspired to surgery, but has contented himself with the study and practice of massage. He claims that John Beveridge was the pioneer in this work, and that Scotland was its birthplace. Chapters on rheumatism, gout, paralysis, neurasthenia, liver, stomach and intestinal complaints, fractures and dislocations, spinal curvature, sprains, and osseous growths, are illustrated by numerous successful cases. We are not told anything of the unsuccessful attempts. The utility of the finger-tips, guided by education and experience, has long since passed the stage of scepticism and discussion.

**Bath Waters: A Rational Account of their Nature and Use, with special reference to Gout, Rheumatism, and Rheumatoid Arthritis.** By Preston King, M.D. With an Historical Sketch by [the Rev.] S. Baring-Gould, M.A. Pp. 128. Bristol: J. W. Arrowsmith. [n.d.]—This is a pleasantly-written popular account of the diseases which are treated at Bath, and of the methods employed there. It can hardly be said to fill a gap or supply a want; for we are already indebted to many successful Bath practitioners for similar works. Whilst the descriptions of treatment may be trusted, the discussions of the pathology of the diseases can hardly be said to be illuminating, nor are the author's conclusions to be implicitly relied on.

**Illustrations from "First Aid" to the Injured and Sick.** By Drs. Warwick and Tunstall. Bristol: John Wright & Co.—A series of sixteen sheets of diagrams, each 36 by 26 inches, all fixed on a metal roller suitable for hanging on the wall to illustrate "First Aid" lectures. The sheets devoted to anatomy go too much into detail, and in those which show the various methods of using triangular and roller bandages fewer diagrams on each sheet would have given greater clearness. The diagrams will no doubt be useful to lecturers.

**Infant Feeding in its Relation to Health and Disease.** By Louis Fischer, M.D. Pp. viii., 359. Philadelphia: F. A. Davis Company. 1901.—This is a somewhat elaborate, not to say complicated, treatise on infant feeding; full of references to papers which have been published on the subject. The general impression produced is one of a lack of precise direction as to what to do in any particular case. Thus, under the treatment of rachitis, we find "Rachitic children require milk, meat and eggs; plenty of cereals, like wheat, barley, rice, farina, sago, oatmeal, hominy; they require butter, and if they will not take butter, then cod-liver oil or liparina; iron will be found valuable, as well as Fellow's hypophosphites; malt extract and ferrum lacticum are indicated." This is in striking contrast to the plain and simple teaching of Cheadle, which is followed with so much success in this country.

**A Handbook of Diseases of the Nose and Pharynx.** By JAMES B. BALL, M.D. Fourth Edition. Pp. xii., 439. London: Baillière, Tindall and Cox. 1901.—Some necessary changes have been made in this book, particularly in the chapter treating of diseases of the accessory sinuses, but the author has suceeded in presenting, within a moderate compass, such an account of the symptoms and treatment of diseases of the nose and pharynx as will be useful to the practitioner and student. The book is pleasantly written and is sensible and practical, being entirely free from the crazes which so much disfigure the writings and sayings of specialists in nasal surgery.

**A Treatise on the Diseases of the Ear.** By T. MARK HOVELL. Second Edition. Pp. xxvi., 808.—London: J. & A. Churchilll 1901.—In our review of the first edition of this work we praised its thoroughness and completeness. Much new matter has been added in the present edition, and many of the chapters have been almost entirely re-written. The work as it now stands is thoroughly modern, and we have pleasure in strongly recommending it to those specially interested in diseases of the ear and in the associated affections of the naso-pharynx. The reader will find a very wide range of therapeutic resources for the relief of ear diseases judiciously noticed, and full directions given for the application of those which have best stood the test of long trial. Unfortunately, success in the treatment of these diseases, as in that of others, is often inversely proportional to the number and variety of the remedies which have been, and are, employed for them. Further progress in the treatment, for example, of chronic adhesive catarrh would seem more likely to be made in the direction of prevention than in that of adding to the number of the already numerous but usually ineffective remedies which are applied after the disease has produced its more prominent ill effects. We have not noticed any mention of the use of the electrically-driven *masseur* in the treatment of the above affection, though the instrument seems to be more efficacious in many cases than the hand instruments. Also the use of Eustachian injections of sterilized liquid vaseline seems not to have been noticed. We cannot agree with the author that "in most cases it is necessary to snare off the posterior extremity of each inferior turbinate body" when post-nasal adenoids require removal. The author states that he has systematically done this for some years past. It is interesting to note that modified Löwenberg's forceps are recommended for the removal of adenoids, rather than curettes. Of the curettes, Delstanche's modification with the hinged box and hooks which catch the growth is preferred by the author, and he usually operates with the patient supine, and the head, but not the shoulders, raised. In alluding to the value of the detection of a cord-like swelling in the position of the upper part of the jugular vein as a sign of thrombosis of the sigmoid sinus, the ambiguity which often arises from the fact that inflamed glands may cause a swelling in the same region is not mentioned.

**Lessons on Massage.** By MARGARET D. PALMER. Pp. xiv., 234. London: Baillière, Tindall & Cox. 1901.—This little book is well written, and should prove very useful in the hands of nurses and beginners generally. Although it does not contain anything new or original, it deals with the methods of treatment in a very practical manner, giving a good description of the massage movements and of the exercises suitable in different cases. In this last respect it has an advantage over most books on massage. A large portion of it is devoted to anatomy and physiology. The illustrations are numerous and good.

**A Text-Book of Medicine.** By Dr. ADOLF STRÜMPELL. Third American Edition. Translated from the Thirteenth German Edition by HERMAN F. VICKERY, M.D., and PHILIP COOMBS KNAPP, M.D. With Editorial Notes by FREDERICK C. SHATTUCK, M.D. Pp. xxii., 1242. London: H. K. Lewis. 1901.—This is the third American, translated from the thirteenth German edition, of this widely-known and popular text-book, which the author tells us has been translated into most European languages and into Japanese. This being the case, it is not necessary for us to say more than that the work has been thoroughly revised, and in the author's words brought "up to the level of contemporary medical thought and knowledge." The work is undoubtedly one of the best text-books on medicine to be placed in the hands of the student or medical graduate; its distinguishing and valuable feature being, perhaps, the consistent keeping in view of the inter-dependence of clinical medicine, pathological anatomy and general pathology. We do not expect from nor desire in a text-book exhaustive descriptions of rare maladies, but no essential points in our present knowledge of disease are omitted from consideration in this volume. The American editor and translators have made valuable additions to the text throughout the book, which appear in nearly every chapter; occasionally, as on the use of stimulants in pneumonia, they do not hesitate to express dissent from the author's views. These additions certainly enhance the value of the book to English readers. A chapter on Plague has also been added by the translators, and in our opinion the favourable estimation in which the book has been held in the past will be maintained in the future by this new edition.

**Some Landmarks in the Progress of Medical Science**: being the Annual Oration at the Medical Society of London. By F. Richardson Cross, M.B. Pp. 43. London: Harrison and Sons.—This address, delivered on May 20th, 1901, is an excellent retrospect of some of the great periods and of the principal actors in the course of the development of the science of medicine from the time of the early Egyptians down to the close of the Victorian era. It must have been a Herculean task, but the result is well worthy of the labour and cannot fail to be of interest and profit to those who peruse it.

**A Short Practice of Midwifery.** By Henry Jellett, M.D. Third Edition. Pp. xxv., 533. London: J. & W. Churchill. 1901.—This book is practically a description of the treatment adopted in the Rotunda Hospital, Dublin, and the fact that it has already reached the third edition is sufficient evidence of its popularity. The first two editions of the work were intended to serve as a handbook for nurses, as well as for students and medical practitioners; but we are glad to find that in the present edition this unsatisfactory arrangement has been altered, and a shortened edition prepared for the use of nurses. In the chapter on Obstetrical Diagnosis the author strongly recommends the substitution of abdominal for vaginal examination whenever possible. The importance of asepsis in midwifery is strongly insisted upon. Dr. Jellett condemns routine douching both ante-partum and post-partum, but lays down some very practical rules for its use under special circumstances. In accidental hemorrhage he recommends the use of the vaginal plug in all cases in which the membranes are intact. Although this treatment is at variance with the rules laid down in most text-books, its adoption at the Rotunda has been followed by very good results. The various theories as to the causation of eclampsia are fully considered, and in discussing its treatment he condemns the use of chloroform on account of its liability to cause death from heart failure. In cases where sedatives are necessary he recommends the hypodermic injection of morphia. With the object of removing toxic substances from the blood, he advocates the intra-cellular or intra-venous injection of saline solution. There are two very useful chapters on the management of the infant, and the common diseases to which it is liable. The text throughout is clear, and the value of the work increased by a number of excellent illustrations.

**Studies in the Psychology of Sex: Sexual Inversion.** By Havelock Ellis. [Second Edition.] Pp. xi., 272. Philadelphia: F. A. Davis Company. 1901.—We much regretted the original publication of this book through certain channels which later on obtained for it a by no means enviable notoriety, and, no matter what the reason, we are glad to see its second edition issued under more suitable auspices. But it is with distaste that we note in exaggerated form the author's indulgence in profuse detail where the clinical records are concerned. We do not impugn Mr. Ellis's honest intention to provide us with complete pictures of his cases and to do no more than this, and further we accredit him with the ability born of such true purpose to imbue the reader to no slight extent with his own calm and judicial spirit. Yet, the mass of moral garbage that he has accumulated in the form of case histories is distressing, and we emphatically opine, superfluous. It cannot be admitted that the publication of a veritable host of foul imaginings serves a sufficiently necessary end. Truly, it is the business of an author to examine every detail, marshal every fact, then to obtain for his readers a filtrate of his investigations, but, especially in a subject of this nature, nothing is gained by placing these elaborations before the public unless demanded in direct support of some fact or principle. We see no evidence that such a record as Case 26 for instance, occasioning as it does nearly twelve pages of small type, is justified by its results. It is not to be supposed that the circulation of this book is confined to medical men, nor is there weighty reason why, if conceived permissible for them, it should be prohibited to any reader of maturity and discretion, not prompted to its perusal by a spirit of mere prurient curiosity. But a certain number of copies must find their way into the hands of persons unfit to properly appreciate the contents, and in its present form we consider this likelihood most unfortunate. At the same time to mark our sense of belief in Mr. Ellis's integrity of purpose, we can affirm that had he chosen to eliminate unnecessary clinical description (as we think), and to have expunged a most regrettable appendix in defence of sexual inversion, for which his pen is not responsible, we would have welcomed one work which, amongst many unworthy congeners, advanced in scientific spirit our knowledge of sex psychology.

**Index-Catalogue of the Library of the Surgeon-General's Office, United States Army.** Second Series. Vol. VI. G—Hernette. Pp. 1051. Washington: Government Printing Office. 1901.—Like its predecessors, the present volume bears witness on every page to the painstaking care bestowed on its preparation. It includes 15,589 author-titles, 5,962 subject-titles of books and pamphlets, and 30,561 titles of articles in periodicals. The work is absolutely indispensable to every library, and no praise is too great for it.

**Tendencies to Consumption: how to Counteract them.** By J. D. E. Mortimer, M.B. Pp. viii., 138. London: The Scientific Press, Limited. [n.d.]—This is not a record of any original work, but it is a concise summary of the nature of tubercular disorders and of the consequent steps to be taken against their development. It is intended as an aid to the practitioner, rather than for self-glorification, on the principle that directions are likely to be put aside as of little importance unless some intelligible reasons are given for their enforcement. The author gives very sensible advice on all questions of hygiene relating to the pre-tubercular state.

**Elements of Practical Medicine.** By ALFRED H. CARTER, M.D., M.Sc. Eighth Edition. Pp. xvi., 590. London: H. K. Lewis. 1901.—There would have been no need for an eighth edition if the book had not proved its adaptation to the wants of the age. Good type, easy to read, condensed and definite in its statements, no diffuse verbosity, and accuracy,—these are qualities which have commended it to the student. The tables of diagnosis are particularly clear and useful; doubtless the therapeutic index is convenient to the uninitiated, but the practitioner should be able to carry principles into practice without the unnecessary burden of a multitude of formulæ.

**Golden Rules of Aural and Nasal Practice.** By PHILIP R. W. DE SANTI. Pp. 87. Bristol: John Wright & Co. [N.D.]—We question the usefulness of works of this class. The facts are for the most part correctly stated, but they are strung together in a collection of disjointed sentences, which are very unreadable and of very little use for reference.

**Pulmonary Tuberculosis: its Prevention and Cure.** By CARLO RUATA, M.D. Pp. vii., 143. Bristol: John Wright & Co. 1901.—The professor of Perugia gives an excellent analysis of the anatomical, physiological, pathological, etiological, and therapeutic facts which relate to tuberculosis of the lungs, as a preliminary to an exposition of his belief that the patient does not die of pure tuberculosis, but always in consequence of the infection of the lung lesions by atmospheric micro-organisms, and that the only rational principle by which to treat tuberculosis is the same which guides the surgeon in the antiseptic treatment of wounds. Continuous inhalations form the basis of his treatment, and from these he has obtained good results. He prefers "pure air," obtained by wearing a mask, to the simple open-air method now commonly adopted.

**The Cure of Pulmonary Tuberculosis.** By Prof. VINCENZO CERVELLO. London: B. & W. Cohen.—This pamphlet of 28 pages is the substance of communications made to the Royal Academy of Medical Science, in Palermo, in April, 1899. It relates to the inhalation of formaldehyde. A medicated atmosphere is obtained by means of a special apparatus called the "Salus Vaporiser," and the substance from which the vapour is derived is called "Igazolo." From these remedies the author has great expectations, but the clinical evidence is not sufficient to form a basis for any deductions therefrom.

**A Retrospect of Surgery during the Past Century, being the Hunterian Oration of the Hunterian Society, 1901.** By JOHN POLAND. Pp. 97. London: Smith, Elder & Co. 1901.—The many advances of surgery during the nineteenth century are ably passed in review, and though the limit of a Hunterian oration must necessitate an abbreviated treatment of so wide a subject, the author has maintained a due proportion in its various parts. The pages of this brochure form a fitting commentary to those who unwisely thought with Boyer that "surgery seems to have attained the highest degree of perfection of which it is capable."

**Transactions of the Obstetrical Society of London.** Vol. XLI. London: Longmans, Green, and Co. 1900.—This volume contains many interesting records of cases and papers. We notice among the curiosities two cases in which tumours were expelled from the uterus during natural labour or removed immediately afterwards. In one case Arnold Lea reported the expulsion of a tumour which, on examination, was reported to be probably an acardiac fœtus in a rudimentary condition. Dakin reported the enucleation of a tumour and its removal from the uterus immediately after labour, which proved to be a fibroid. These cases, although rarities, might give rise to great difficulties and their occasional occurrence should be borne in mind. Dr. Lediard reports the case of a woman who evacuated per rectum the bones of a fœtus of six or seven months developed from an ectopic gestation sac. The gestation seems to have occurred in August, 1893, and bones began to to be passed in September, 1894, the sac being apparently emptied by February, 1895. This case is a striking instance of the resistance to mortality occasionally met with. Dr. John Phillips contributes an interesting case of fatal acute peritonitis, without discoverable cause, complicating pregnancy and labour.

**Transactions of the American Association of Obstetricians and Gynæcologists.** Vol. XIII. Philadelphia: Wm. J. Dornan. 1901.—The first thing which strikes us in the perusal of this volume is the publication under the above title of such papers as those on gastric ulcer and gallstones. We thought at first that the obstetricians and gynæcologists in America, from long concentration of their attention on diseases of women, had forgotten that these diseases do sometimes occur in man, and had even traced a connection between irregularities of menstruation and the formation of gallstones and the liability to gastric ulcer. But this explanation is clearly erroneous, for on reference to the papers we find that some of the patients referred to were men. The explanation clearly is, that the gynæcologist in America is so largely an abdominal surgeon, that he is apt to forget that he is a gynæcologist at all, and it is quite immaterial to him even of what sex his patient may be. However, the volume contains numerous papers on various diseases of women. We are surprised not to find any on obstetric subjects in the proceedings of a society of both obstetricians and gynæcologists. Many of the papers will prove of interest to gynæcologists. The illustrations are remarkably good.

**Transactions of the American Pediatric Society.** Vol. xii. Reprinted from *Archives of Pediatrics.* 1900.—It is impossible to touch even briefly upon the numerous interesting papers in these *Transactions*, but a few points of interest or importance may be referred to. In a careful experimental paper upon the pancreatic digestion of casein, Dr. Rachford comes to the conclusion that lime water is of more value than a decoction of one of the cereals in aiding pancreatic digestion. Dr. Fruitnight records a case in which a large post-otitic abscess situated in the left temporao-sphenoidal lobe had been associated during life with amnesic aphasia. Dr. Edward P. Davis considers that the treatment of hydrocephalus by craniectomy is of little value. An epidemic of paralysis in children is recorded by Dr. Dwight Chapin. One child, which died, showed in the spinal cord the characteristic lesions of infantile paralysis, but the majority of the cases completely recovered. In others wasting of some muscles remained. Dr. Chapin thinks that in those which recovered possibly the peripheral nerves suffered, but the spinal cord escaped. A large number of cases of examination of the blood in infancy are recorded by Stengel and White, and amongst other cases of special interest or rarity which are recorded is one of hemorrhage into the suprarenal capsule, by Hamill; one of congenital disease of the left side of the heart, by Cotton; and a case of general subcutaneous emphysema following coughing is by Cotton. In a paper upon enteric fever, by Blackader, it is stated that below the age of fifteen the duration of the fever in the great majority of cases is under three weeks. The onset is, however, more sudden than in adults. In only eight out of 100 cases a resemblance to Wunderlich's ascent was noted.

**Elementary Bandaging and Surgical Dressing.** By the late Walter Pye. Revised by Thos. Carwardine. Ninth Edition. Pp. viii., 214. Bristol: John Wright & Co. 1901.—We are glad to welcome another edition of this little volume, which has proved so useful to students and nurses for the past sixteen years. The general arrangement of the book is unaltered. The illustrations have been added to and improved.

**The Causes of Death among the Assured in the Scottish Widows' Fund and Life Assurance Society, from 1874 to 1894 inclusive.** Reported by Claud Muirhead, M.D. Pp. viii., 103. Edinburgh: R. & R. Clark, Limited. 1902.—This analysis of the causes of death amongst the assured during a period of twenty-one years cannot fail to be both interesting and instructive. The author's comments on cancer and consumption are of especial importance. As regards cancer he summarises the following conclusions:—

"1. The registered increase in the number of deaths from cancer is undoubted. This is proved by our own statistics, and corroborated by all other authorities.

"2. After allowing that this increase is not wholly real, but may be accounted for, to some extent, on the assumption that the true nature of obscure cases of malignant disease has been recognised with ever increasing certainty in recent years, and that, as a consequence, the statement of death has been made with greater precision than had been formerly the case, there remains a large real increase to account for the large and progressive mortality from this disease.

"3. The age period at which death from cancer is most frequent is gradually declining, according to the Scottish Widows' Fund returns.

"4. The average age at death from cancer among our members declined by two years from 1874–80 to 1888–94, as contrasted with a rise in the average at death from all causes of a little over two years.

"5. The office returns mark a *decrease* in deaths from *internal* cancer of 7.70 per cent., and an *increase* in deaths from *external* cancer of 18.89 per cent. of the percentages in the first septennium as contrasted with the third."

A consideration of the death-rate from consumption shews:—

1. That the death-rate from consumption among the general population decreased 16 per cent. in 1881–90 as compared with 1871-80.

2. That the death-rate among the members of the Scottish Widows' Fund:—

*a.* Decreased 15 per cent. from 1874–80 to 1881–87.

*b.* Decreased 27 per cent. from 1874–80 to 1888–94.

An examination of the tables shews that the prevalence of consumption is by no means confined to persons under middle life, and contradicts the popular belief that this disease belongs to the period of youth and early manhood. They also shew that 15 per cent. at least of proposers to the Society for assurance, and of those accepted by the Society, will shew a record of death by consumption among their parents. We are not entitled, because too much has been made of this inherited vulnerability in the past, wholly to ignore it, and to treat it as a negligible quantity.

**Kitchen Physic.** By W. T. Fernie, M.D. Pp. xxviii., 596. Bristol: John Wright & Co. 1901.—It has been said truly, "There is no question more frequently asked, or which a doctor has more difficulty in answering to the satisfaction of himself and his patients, than 'What do you wish me to eat?'" And, as the author states in his preface, "all valid reasons have ceased to exist why most persons of ordinary intelligence should not gain the ability to solve such a question for themselves by learning the first principles which underlie kitchen physic and are plain of comprehension." There is in this volume a great deal of much practical value to the lay reader, and much too of academic interest, for the writer has the art of writing attractively on commonplace topics. For example, we find that the pathology and the general lines of treatment of boils and carbuncles are plainly stated, and lead up to the dietetic indications and contra-indications and the use of various applications such as fomentations. Under the head of "Cancer" the question of various articles of food being a possible source of the disease is discussed, and under "Diarrhœa and Dysentery," "Digestion and its Affections," "Dropsy," "Gout," and so on, the suitable diet is carefully laid down. But the author has enhanced the value of his work by references to the dietetic methods of the German, French, and other nationalities, while his copious extracts from the writers of past times bear witness to the untiring labour and wide research of the author and add much to the interest of the subject.

**The Sanitary Inspector's Handbook.** By ALBERT TAYLOR. Third Edition. Pp. xvi., 408. London: H. K. Lewis. 1901.—In this third edition Mr. Taylor has, by careful additions and revision, maintained the useful character of the book. We are particularly satisfied with the short notes (p. 209) on infectious diseases, containing just what an inspector, who is not a doctor, requires to know; and are glad to find that these have been quoted from a reliable source, and afford really useful information—a statement which cannot be made about all recent text-books.

**Syllabus of Lectures to Nurses.** By ANDREW DAVIDSON, M.D. Pp. 101. London: Scientific Press, Limited. [N.D.].—This little book is exactly what its name indicates. It is divided into five parts: Anatomy; Physiology; First Aid; The Care of the Sick; and The Care of the Insane. It is carefully arranged in headings and is merely a list of chief points which have to be enlarged on by a lecturer. There is nothing in it to criticise. It will save anyone about to lecture to nurses a considerable amount of trouble in setting out the lines which one should take in giving an elementary course. It should also prove useful to nurses in giving them ideas as to what to look up in other books; beyond this it has no scope and is not meant to have.

**Annual Report on the Year 1901.** E. MERCK, Darmstadt.—The fifteenth volume of this instructive report was published in March, 1902. It contains a concise summary of recent pharmacological investigations. At the commencement there is a lengthy article of twenty-five pages, giving the results of investigations on opium tests. Other articles of interest are those on cacodylates, and various dietetic preparations. The latest results obtained by the newest and rarest of drugs are here to be found. The report is full of interest and is compiled with great judgment.

